# Non-base-contacting residues enable kaleidoscopic evolution of metazoan C2H2 zinc finger DNA binding

**DOI:** 10.1186/s13059-017-1287-y

**Published:** 2017-09-06

**Authors:** Hamed S. Najafabadi, Michael Garton, Matthew T. Weirauch, Sanie Mnaimneh, Ally Yang, Philip M. Kim, Timothy R. Hughes

**Affiliations:** 1 Department of Human Genetics, McGill University, Montreal, QC Canada; 2 McGill University and Genome Quebec Innovation Centre, Montreal, QC Canada; 3 Donnelly Centre for Cellular and Biomolecular Research, University of Toronto, Toronto, ON Canada; 4 Center for Autoimmune Genomics and Etiology, and Divisions of Biomedical Informatics and Developmental Biology, Cincinnati Children’s Hospital Medical Center, Cincinnati, OH USA; 5 Canadian Institute for Advanced Research, Toronto, ON Canada; 6 Department of Computer Science, University of Toronto, Toronto, ON Canada; 7 Department of Molecular Genetics, University of Toronto, Toronto, ON Canada

## Abstract

**Background:**

The C2H2 zinc finger (C2H2-ZF) is the most numerous protein domain in many metazoans, but is not as frequent or diverse in other eukaryotes. The biochemical and evolutionary mechanisms that underlie the diversity of this DNA-binding domain exclusively in metazoans are, however, mostly unknown.

**Results:**

Here, we show that the C2H2-ZF expansion in metazoans is facilitated by contribution of non-base-contacting residues to DNA binding energy, allowing base-contacting specificity residues to mutate without catastrophic loss of DNA binding. In contrast, C2H2-ZF DNA binding in fungi, plants, and other lineages is constrained by reliance on base-contacting residues for DNA-binding functionality. Reconstructions indicate that virtually every DNA triplet was recognized by at least one C2H2-ZF domain in the common progenitor of placental mammals, but that extant C2H2-ZF domains typically bind different sequences from these ancestral domains, with changes facilitated by non-base-contacting residues.

**Conclusions:**

Our results suggest that the evolution of C2H2-ZFs in metazoans was expedited by the interaction of non-base-contacting residues with the DNA backbone. We term this phenomenon “kaleidoscopic evolution,” to reflect the diversity of both binding motifs and binding motif transitions and the facilitation of their diversification.

**Electronic supplementary material:**

The online version of this article (doi:10.1186/s13059-017-1287-y) contains supplementary material, which is available to authorized users.

## Background

**Fig. 1:**
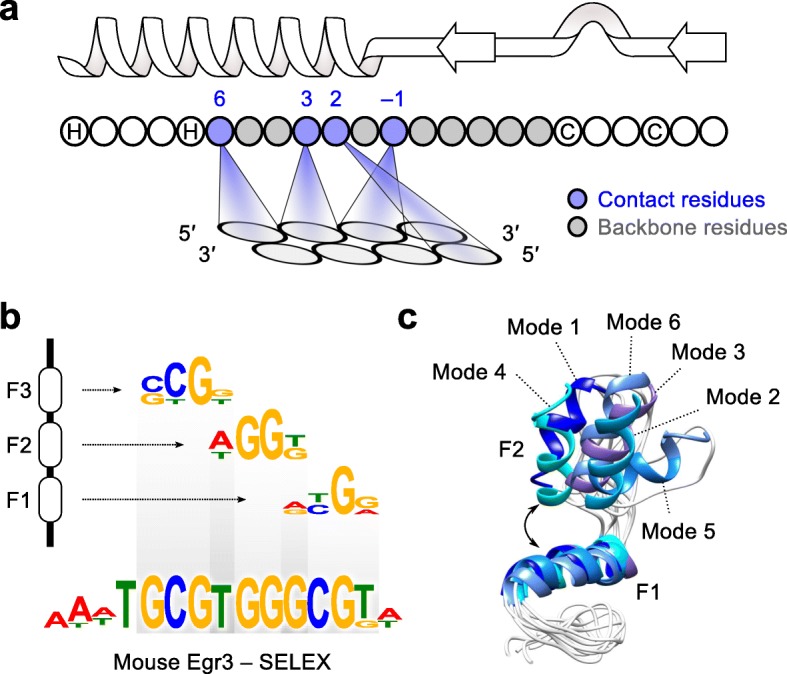
DNA sequence recognition by the C2H2-ZF domain. **a** *Schematic representation* of C2H2-ZF structure and interaction with DNA. The base-contacting residues (*blue*) interact with three or four DNA bases. The eight non-base-contacting residues of the zinc finger (bounded by C and H) are shaded *gray. Arrows*, *loop*, and *helix* in the schematic illustration at the top denote the beta-sheets, turn, and alpha helix structures, respectively, with the *arrowheads* pointing toward the protein C-terminus. **b** Example of motifs obtained from individual C2H2-ZF domains using B1H [16] and comparison to the motif obtained from full-length protein using SELEX [45]. **c** Neighboring C2H2-ZFs can interact with each other and affect the orientation of binding, resulting in multiple binding modes depending on the residues at the interface of the interaction. Figure is adapted from ref [7]

Transcriptional regulatory programs are thought to evolve primarily by alterations to *cis*-regulatory sequence [1], while *trans*-regulators are often highly conserved [1–3]. The metazoan C2H2 zinc finger (C2H2-ZF) family of transcription factors (TFs) represents a striking exception. C2H2-ZF proteins generally consist of tandem C2H2-ZFs, each of which contacts three or more DNA bases, with the sequence preference of each C2H2-ZF controlled mainly by four base-contacting residues in its alpha helix [4–6] (Fig. 1a). The DNA sequence preferences of full-length C2H2-ZF proteins typically resemble a concatenation of the preferences for the individual domains (Fig. 1b), often modified by interactions between tandem C2H2-ZFs, which can be modulated by residues at the interface between C2H2 domains [4–8] (Fig. 1c). Genome sequencing has revealed independent expansions of C2H2-ZF-containing family members in different taxa, with frequent rearrangement of C2H2-ZF domains as well as evidence for positive selection on the base-contacting residues in some cases [9–14]. These changes can lead to new DNA preferences with dramatically different genome-wide binding landscapes and new functions [15].

The C2H2-ZF proteins show the largest expansions in metazoans and undertake a wide variety of regulatory functions in these organisms [16]. The typical metazoan genome contains ~280 C2H2-ZF proteins and a total of ~2000 C2H2-ZF domains (Additional file 1: Figure S1), making it the most numerous protein domain in many metazoans, including humans [12, 17, 18], as well as the largest class of TFs by far [19, 20]. The extent to which diversification globally impacts the sequence specificity of C2H2-ZF domains remains unclear, however. It is also unknown why or how metazoan C2H2-ZFs differ so dramatically from their non-metazoan counterparts in their ability for diversification and whether there are differences among metazoan C2H2-ZFs in this regard.

Here, we undertook a systematic examination of the sequence preferences of C2H2-ZFs across the eukaryotes. We focused on individual C2H2-ZF domains because they are highly modular in nature, while their frequent rearrangement complicates evolutionary analysis of whole proteins. Our extensive survey and evolutionary reconstructions show that virtually all metazoans possess a nearly complete “vocabulary” of individual C2H2-ZF domains. The extant C2H2-ZF domains typically do not bind the same sequences as their progenitors at the base of eutheria, however. We also identify fundamental biochemical differences between metazoan and non-metazoan C2H2-ZFs, in which non-base-contacting residues contribute to diversification in metazoan by favoring a larger degree of hydrogen bonding. We provide evidence that these differences facilitate the rapid evolution of C2H2-ZF sequence preferences, particularly in the eutheria, and introduce the term “kaleidoscopic” to describe their extensive and facilitated diversification.

## Results

### Metazoan-specific diversification of C2H2-ZF binding vocabulary

We began with a global analysis of the DNA sequence preferences of individual C2H2-ZF domains across 283 eukaryotes. We utilized a recently described recognition code that uses the identity of the base-contacting residues to map the nucleotide preference of each C2H2-ZF at each position of its binding site [16]. This recognition code is trained to predict the DNA sequence preferences of individual C2H2-ZFs when they are fused to the F1–F2 region of mouse Egr1 protein. Neighbor effects can impact the sequence preferences of individual C2H2-ZFs on sequence specificity [4-8], but we previously showed that the recognition code provides a good general approximation of the sequence preferences of individual C2H2-ZFs in their natural context (median Pearson correlation of 0.70 between predicted and experimental motifs [16]). Among ~142,000 unique eukaryotic C2H2-ZFs in total, we focused on 106,771 C2H2-ZFs that have the canonical length of 23 amino acids, to avoid complexities that might arise from variable lengths (i.e. insertions and deletions) in subsequent analyses.

**Fig. 2:**
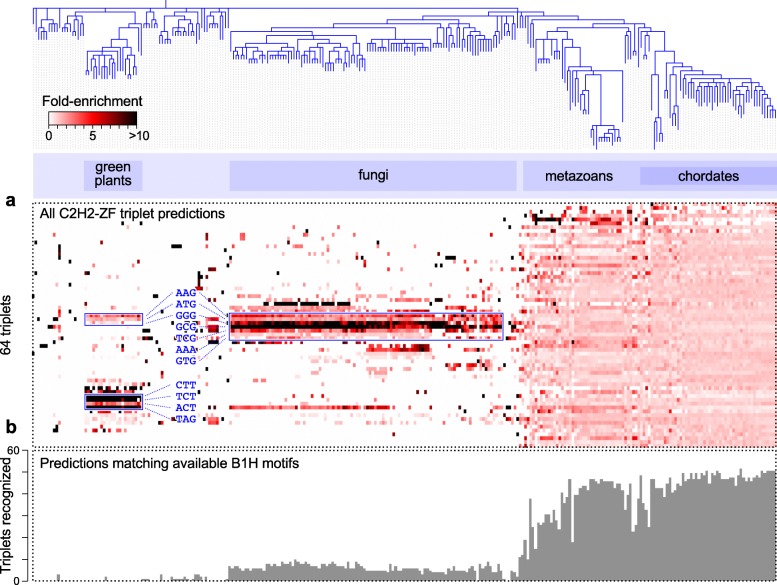
Overview of sequence preferences of individual C2H2-ZF domains. **a** The *heatmap* shows the ratio of C2H2-ZFs with highest preference for each DNA triplet (*y-axis*) in each organism, relative to the count expected by chance (the expectation is calculated as the sum of counts in the column multiplied by the sum of counts in the row divided by the total sum of counts). Species tree (*x-axis*) is from the NCBI taxonomy database [27]. *Y-axis* is clustered. Fungi- and plant-specific DNA triplets are labeled. C2H2-ZF counts can be found in Additional file 1: Figure S2. **b** Number of triplets recognized by at least one C2H2-ZF in different organisms. Only C2H2-ZFs with matching predicted and experimental motifs are included, i.e. C2H2-ZFs whose base-contacting residues match at least one experimentally examined C2H2-ZF (by B1H [6, 16]) and whose recognition code-predicted motif matches the experimental motif (Pearson correlation > 0.9)

Our first objective was to compare the C2H2-ZF motif repertory of different genomes. To do this, we associated each C2H2-ZF with its most highly preferred DNA triplet, as previously described [8], and counted the number of C2H2-ZFs binding each triplet in each genome (Fig. 2a and Additional file 1: Figure S2). In most metazoans, almost all DNA triplets are included in the collective binding vocabulary of C2H2-ZFs, even considering only those with highest specificity and with canonical interface residues (Additional file 1: Figure S2) or those with available B1H data for a C2H2-ZF with identical base-contacting residues (Fig. 2b). Most vertebrates contain > 24 C2H2-ZFs that recognize a typical DNA triplet (Additional file 1: Figure S3). In contrast, non-metazoan C2H2-ZFs, which are much fewer in number, are largely biased toward a small subset of DNA triplets.

**Fig. 3:**
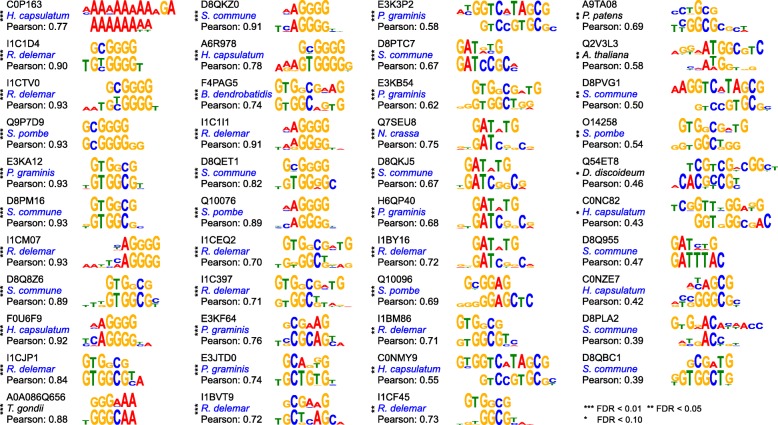
Predicted and PBM motifs for 43 non-metazoan C2H2-ZF proteins. The proteins are identified by their UniProt IDs. For each protein, the recognition code-predicted motif is shown on top and the PBM motif is shown below the predicted motif. Fungal organisms are shown in *blue. Asterisks* represent the significance of the Pearson similarity of the PBM and recognition code-predicted motifs, calculated as previously described [16]

**Fig. 4:**
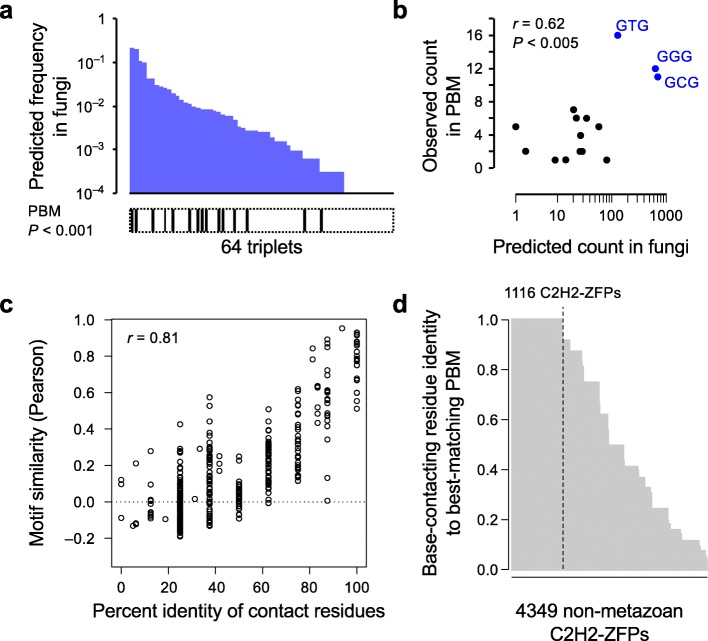
Limited diversity of non-metazoan C2H2-ZF proteins. **a** Triplets that appear at least once in the PBM motifs of fungal proteins (*black bars* at the bottom) are among those with the highest predicted frequencies based on the recognition code (Mann–Whitney U test *P* < 0.001). The *x-axis* represents the 64 triplets, sorted by their predicted frequency. **b** Correlation of the predicted and observed frequencies for DNA triplets that are observed at least once in the PBM motifs. The top three triplets with the highest predicted and observed frequencies are highlighted in *blue*. **c** Percent identity of contact residues between pairs of proteins is highly predictive of similarity of their motifs. Each *dot* represents one pair of non-metazoan C2H2-ZF proteins, with the *x-axis* corresponding to the similarity of the entire set of their contact residues and the *y-axis* showing the similarity of their motifs as determined by PBMs (motif similarity was measured as correlation in predicted affinity to different sequences, as previously described [46]). C2H2-ZF proteins with 100% identical base-contacting residues have motif similarity of 0.76 ± 0.12. **d** Of 4349 non-metazoan C2H2-ZF proteins that have at least two C2H2-ZF domains, 1116 have base-contacting residues that are identical to at least one of the 43 proteins we analyzed by PBM and therefore recognize motifs that are nearly identical to their matching PBM motifs

To ensure that this bias in the binding vocabulary of non-metazoan C2H2-ZFs is not an artefact of the computational prediction of binding sequences, we selected 43 diverse non-metazoan C2H2-ZF proteins (including 39 from fungi, which are the most numerous) and examined their binding preferences by protein-binding microarrays (PBMs). The majority of the PBM motifs were highly similar to the predicted motifs (37/43 at false discovery rate < 0.05, Fig. 3), with DNA triplet frequencies that were observed in the PBM motifs largely in agreement with those predicted by the recognition code (Fig. 4a, b).

The proteins we analyzed by PBM were chosen to include diverse base-contacting residue combinations (30 unique combinations), as well as several proteins with identical base-contacting residues, but different non-base-contacting residues (19 proteins match at least one other protein in the base-contacting residues, but are < 70% identical at non-base-contacting residues). The PBM results suggest that, for non-metazoan C2H2-ZF proteins, having identical base-contacting residues is a strong indication for having nearly identical motifs (Fig. 4c), with slight differences that are potentially due to different base-contacting residue orientations relative to DNA [7, 21]. Assuming this is the general case, these 43 PBM analyses can be used to infer motifs for 1116 C2H2-ZF proteins with matching base-contacting residues, which constitute 25% of all extant non-metazoan C2H2-ZF proteins (Fig. 4d). These observations confirm that the binding motifs of non-metazoan C2H2-ZF proteins have limited diversity and tend to evolve slowly, in stark contrast to metazoan C2H2-ZF proteins [16].

Together, these analyses show: (1) that virtually all metazoan genomes contain a complete or nearly complete set of DNA triplet sequence preferences encoded among their C2H2 domains; and (2) that other lineages (fungi and plants) have a much more limited set of sequence preferences. To our knowledge, these are novel findings. Because these findings are based on computational models, they are approximate. We note, however, that incorrect predictions would mainly add noise to the data, which would not result in the striking trends observed in Figs. 2, 3 and 4. Moreover, we show below that despite potential inaccuracies, these predictions can be used to reveal general mechanisms that underlie diversification of sequence preferences of C2H2-ZFs.

### Reliance on DNA contacting residues for DNA-binding functionality explains the limited vocabulary of non-metazoan C2H2-ZFs

We next sought to determine whether there is some fundamental difference between metazoan and non-metazoan C2H2-ZF domains that could explain why non-metazoan C2H2 domains display such limited diversity in DNA sequence preferences and appear to evolve slowly. We initially examined the sequences recognized. The DNA triplets recognized by non-metazoan C2H2-ZF domains are also recognized by metazoan C2H2-ZFs based on experimental B1H data [16], often using identical base-contacting residues (~88% of base-contacting residue combinations recognize the same triplet or triplets that only differ in one nucleotide between metazoan and non-metazoans, Additional file 1: Figure S4). Thus, the direct sequence recognition mechanism does not appear to explain the rapid evolution of metazoan C2H2-ZF proteins.

We next examined other aspects of the C2H2-ZF domains. As a starting point, we examined the outputs of a computational model we recently described [16] that is aimed at classifying whether a C2H2-ZF binds any DNA sequences, rather than predicting the specific sequence that it prefers. Trained on thousands of natural B1H constructs that score positively in selections for various DNA triplets (vs. thousands that never score positively in any selections), this “DNA-binding functionality model” (here abbreviated as the DBF model) inputs the entire 12 AA sequence between the zinc-binding cysteine and histidine residues into a random forest [22] and outputs an estimate of DBF between zero and one. The DBF score correlates with the highest PBM Z-score obtained for the preferred binding site for C2H2-ZF proteins (Additional file 1: Figure S5a–c), consistent with the notion that C2H2-ZFs with higher DBF scores bind more strongly to specific DNA sequences, as we previously showed that Z-scores correlate with relative affinity [23].

**Fig. 5:**
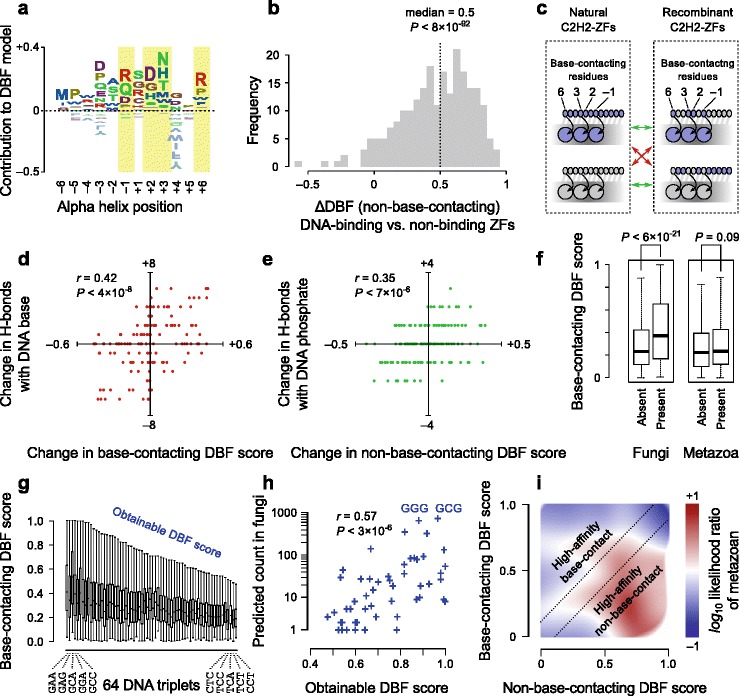
DBF dictates the binding vocabulary of C2H2-ZFs. **a** Contribution of both base-contacting residues (*highlighted*) and non-base-contacting residues to the DBF model. The letter height represents the average value that each amino acid at each position contributes to the DBF score. **b** Evaluation of non-base-contacting residue DBF sub-model. The *x-axis* represents 228 pairs of DNA-binding/non-binding ZFs with identical base-contacting residues [16]. The *histogram* of ΔDBF for 228 pairs of DNA-binding and non-binding C2H2-ZFs with identical base-contacting residues (the non-base-contacting DBF sub-model score of the DNA-binding C2H2-ZF minus that of the non-binding C2H2-ZF). The ZF pairs shown here were excluded from the training set for the full DBF model, which was then deconvoluted to obtain the sub-model that was used for evaluation of performance. **c** *Schematic illustration* of in silico recombination experiments. The base-contacting residues of a natural C2H2-ZF are swapped with those of another C2H2-ZF and both the original and recombinant C2H2-ZFs, in complex with their respective optimal binding sites, are evaluated by structural modeling. The optimal binding site of each C2H2-ZF is determined by PBM [16]. Modeling results are used to reveal the structural basis of the contribution of base-contacting and non-base-contacting residues to the DBF model, based on comparisons showed by *red* and *green arrows*, respectively. **d** *Scatter plot* showing the change in the base-contacting DBF score after mutation of base-contacting residues vs. the change in the number of hydrogen bonds with DNA bases. Both original and mutated base-contacting residues are paired with their respective optimal binding sites for structural modeling. **e** *Scatter plot* showing the change in the non-base-contacting DBF score vs. the change in the number of hydrogen bonds with DNA phosphate backbone. **f** *Tukey boxplot* of DBF scores for base-contacting residue combinations with that are present/absent in fungi C2H2-ZFs (*left*) or in metazoan C2H2-ZFs (*right*). The *P* value corresponds to two-sided Mann–Whitney U test. **g** *Tukey boxplot* of DBF scores for base-contacting residue combinations that bind to each DNA triplet. The greatest DBF score that is within 1.5× the interquartile range of the upper quartile (the upper whisker) is considered the obtainable DBF for each triplet. **h** Obtainable DBF correlates with frequency of C2H2-ZFs that recognize each triplet in fungi. The Pearson correlation of logarithm of count vs. obtainable DBF score is shown. **i** *Heatmap* showing enrichment of metazoan C2H2-ZFs among those with low base-contacting DBF score and high non-base-contacting DBF scores. The distribution of C2H2-ZFs as a function of base-contacting and non-base-contacting DBF scores was estimated for metazoans and non-metazoans separately – the color gradient represents the logarithm of ratio of the probability density function for metazoan C2H2-ZFs relative to non-metazoan C2H2-ZFs

Analysis of the random forests produced shows that the DBF model relies most on residues with previously reported roles in structural stability or interaction with the DNA backbone, such as position +4 of the zinc finger alpha helix with a conserved role in proper folding [24–26] and position +1, which makes contact with the phosphate backbone of the DNA [24]. In addition, however, the DBF model indicates that both the base-contacting and non-base-contacting residues play a role in determining the DNA-binding strength of the C2H2-ZF, since the identity of amino acids at both types of residues is highly informative about the outcome of the DBF model (Fig. 5a).

To explore the possibility that non-base-contacting residues contribute to DNA binding, we deconvoluted the DBF model into two sub-models, one measuring the contribution of the base-contacting residues and the other the contribution of the non-base-contacting residues. These sub-models were produced by re-training the model on only the base-contacting residues (6, 3, 2, –1) or the remaining eight residues. The sub-models output the same score range (0–1) as the original model (Additional file 1: Figure S5d–f). In support of its relevance and accuracy, the non-base-contacting DBF sub-model showed a high degree of accuracy in predicting DNA-binding capability for pairs of C2H2-ZFs with identical base-contacting residue combinations (the better DNA-binding C2H2-ZF was accurately identified in 215 out of 228 C2H2-ZF pairs in held-out B1H data; Fig. 5b).

Computational modeling of protein–DNA complexes revealed a structural basis for both of the sub-models and suggests that the relationship of DBF score to affinity is mediated mainly by hydrogen bonds. In order to separately analyze the impact of DNA contacting and non-contacting residues, we examined in silico mutations in which we exchanged the DNA contacting and non-contacting residues among randomly selected C2H2-ZFs that have experimentally verified motifs [16] (Fig. 5c). We used molecular dynamics to model binding to the optimal binding site for each variant C2H2-ZF (defined as the triplet with the largest PBM z-score [16]). These analyses indicate that the DBF correlates best with the number of hydrogen bonds (r = 0.42, *P* < 4 × 10^–8^ when exchanging DNA contacting residues, Fig. 5d; r = 0.35, *P* < 7 × 10^–6^ when exchanging non-base-contacting; Fig. 5e). Non-base-contacting C2H2-ZF residues primarily formed hydrogen bonds with the DNA phosphate backbone, whereas base-contacting residues formed hydrogen bonds with the nucleotide base, as expected. Other structural characteristics are also reflected in the DBF model; for example, we found a significant correlation between the non-base-contacting DBF score and the possibility for salt bridge formation arising from appropriate Lys and Arg rotamer proximity to DNA PO4 groups (*r* = 0.19, *P* < 0.01, Additional file 1: Figure S6).

The DBF scores for the two sub-models displayed markedly different trends between metazoans and non-metazoans. In particular, the base-contacting residue combinations that appear in fungi mostly correspond to those with higher DBF scores, unlike those in metazoans (Fig. 5f). In fact, we found that the bias in the C2H2-ZF binding vocabulary of non-metazoans appears to be largely explained by the “obtainable DBF score” for each DNA triplet, which reflects the highest DBF score that can be obtained for that triplet, considering only its compatible specificity residues. Obtainable DBF is defined as follows: (1) we first used the recognition code to identify all possible cognate base-contacting residue combinations for each of the 64 DNA triplets; (2) for each DNA triplet we then calculated the DBF score distribution over all possible combinations of cognate base-contacting residues that specify that triplet (Fig. 5g); (3) we then defined the obtainable DBF score for each triplet as the top of the corresponding error bar in Fig. 5g (the error bar represents 1.5× the interquartile range). The DNA triplets that are most commonly recognized by fungal C2H2-ZFs generally have large obtainable DBF, whereas triplets whose recognition requires low-scoring base-contacting residue combinations are rarely recognized by fungal C2H2-ZFs (Fig. 5h). This conclusion is largely insensitive to the definition of the obtainable DBF score with other variations, such as the maximum DBF score for each triplet, also showing significant correlation with the frequency of triplets (*r* = 0.53, *P* < 2 × 10^–5^). We therefore hypothesize that the fungal C2H2-ZFs have limited ability to alter their sequence specificity because they rely on base-contacting residues to achieve high DBF, restricting them to only a select set of base-contacting residue combinations. Consistent with this notion, non-metazoan C2H2-ZFs (most of which are in fungi) typically have low non-base-contacting DBF scores (Fig. 5i).

In contrast, metazoans have a large repertoire of C2H2-ZFs with low-DBF base-contacting residues, which are accompanied by high-DBF non-base-contacting residues as revealed by the sub-models (Fig. 5i). This observation suggests an intriguing mechanism for metazoan C2H2-ZFs to escape the limitation posed by base-contacting residues with relatively poor DNA-binding function: interaction with DNA is enhanced by non-base-contacting residues in metazoan C2H2-ZFs through hydrogen bonds with the DNA phosphate backbone, allowing low-DBF base-contacting residues to be used in the metazoan C2H2-ZF without disrupting the DBF. We therefore further explored the notion that functional robustness provided by non-base-contacting residues may have contributed to the diversity of DNA-binding among metazoan C2H2-ZFs.

### Functionally robust C2H2-ZFs evolve into high-diversity pedigrees

**Fig. 6:**
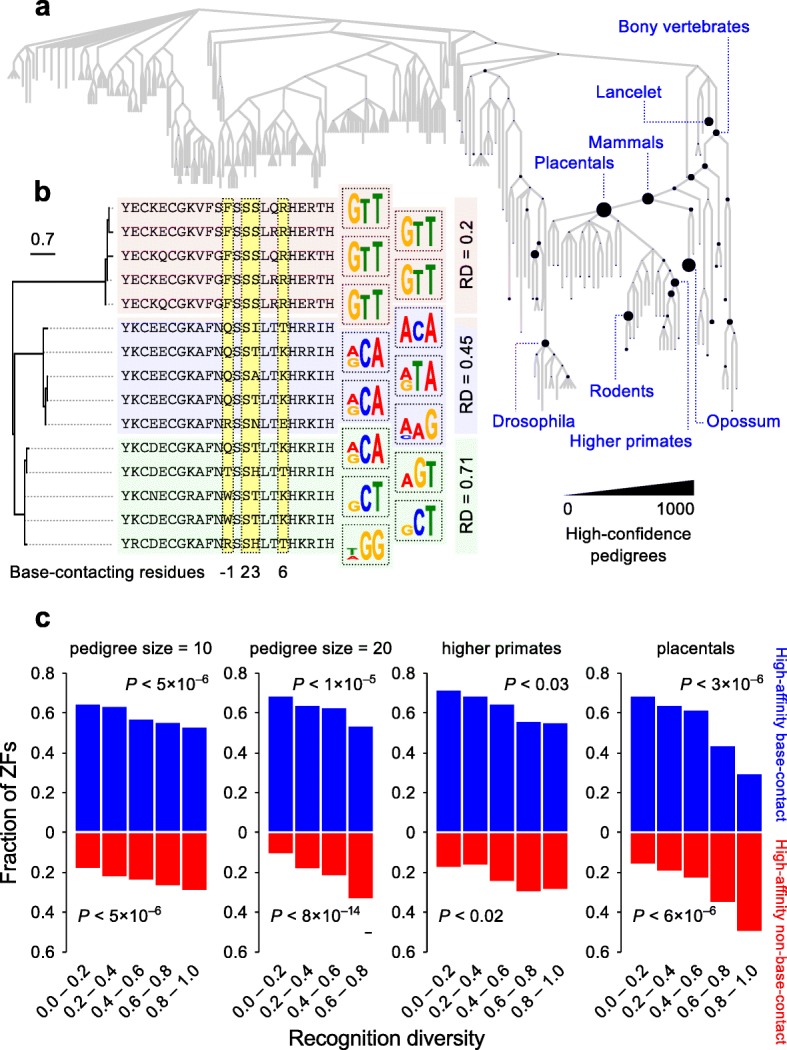
High-confidence pedigrees of C2H2-ZFs reveal the role of functional robustness in evolution of diversity. **a** The species tree of eukaryotic organisms, with the number of high-confidence C2H2-ZF pedigrees that map to each extant or ancestral organism shown by node size. **b** Three example high-confidence pedigrees, each with five extant C2H2-ZFs, with various degrees of binding diversity. The recognition diversity (RD) of each pedigree is shown on the right (see “Methods” for details). All three pedigrees map to the base of higher primates (Simiiformes). **c** Proportion of C2H2-ZFs with high-DBF base-contacting (*blue*) or non-base-contacting (*red*) residues (i.e. DBF difference between base-contacting and non-base-contacting residues > 0.1) within pedigrees of varying recognition diversity, with pedigree selections shown on the top of each panel. *P* values (Mann–Whitney U test) correspond to the difference in the recognition diversities of C2H2-ZFs with high-DBF base-contacting or high-DBF non-base-contacting residues (DBF difference > 0.1) relative to C2H2-ZFs with equally strong base-contacting and non-base-contacting residues (DBF difference < 0.1)

We reasoned that if the non-base-contacting residues contribute to the evolution of diversity in the sequence preferences of C2H2-ZFs, then we should observe a correspondence between non-base-contacting DBF scores and diversity in the sequence preferences of closely related C2H2-ZFs, which are presumably also evolutionarily related. To identify closely related C2H2-ZFs, we first created a maximum-likelihood phylogenetic tree of the 106,771 unique 23 amino acid-long C2H2-ZFs, based on sequence identity of the amino acid residues, excluding the base-contacting residues, which are often under diversifying selection (see “Methods” for details) [9]. While this tree is not a bona fide molecular history, there are 42,880 cases in which the inferred ancestry of an extant C2H2-ZF from maximum parsimony is in perfect accordance with known phylogenetic relationships among species that encode the extant C2H2-ZFs with related sequences [27] (Fig. 6a and Additional file 1: Figure S7a). We refer to these cases as “pedigrees.” The pedigrees cover a wide range of evolutionary divergence times, including numerous instances of recent species-specific expansions in *Branchiostoma floridae* (lancelet) and *Monodelphis domestica* (opossum) (Fig. 6a), KRAB-associated C2H2-ZF expansions in primate and mammalian ancestors (Additional file 1: Figure S7b–d), several thousand C2H2-ZFs that date to the Last Common Ancestor (LCA) of bony vertebrates (Fig. 6a), and ancient pedigrees originating at the base of metazoans. While we identified pedigrees based on individual C2H2-ZFs, the pedigrees are consistent with known orthology relationships between proteins; C2H2-ZFs that are in the same pedigree often form syntenic arrays across orthologous proteins (Additional file 1: Figure S7e and Additional file 2: Table S1).

The expansions observed within pedigrees encompass a wide range of diversification rates in base-contacting residues, including high-diversity pedigrees that can bind to tens of different DNA triplets, as well as pedigrees with largely invariant base-contacting residues. We stratified the pedigrees based on the diversity of DNA triplets recognized by their extant C2H2-ZFs (examples in Fig. 6b, see “Methods” for details) and observed that, irrespective of pedigree size or origin, low-diversity pedigrees mostly consist of C2H2-ZFs with high base-contacting DBF scores (i.e. that presumably rely on their base-contacting residues for obtaining high DNA-binding affinity), whereas high-diversity pedigrees have a consistently higher proportion of C2H2-ZFs with high non-base-contacting DBF scores (i.e. whose interaction with DNA is apparently fortified by non-base-contacting residues) (Fig. 6c and Additional file 1: Figure S8a).

We note that this analysis could potentially be confounded by several factors, including inaccuracies of the recognition code in prediction of the DNA triplets that are preferred by C2H2-ZFs, and the influence of neighboring C2H2-ZFs, which may alter the orientation of binding to DNA and, therefore, affect the sequence preference [18]. Several lines of evidence, however, indicate that the observed correlation between recognition diversity of pedigrees and the fortifying effect of non-base-contacting residues is robust against these factors. First, if the recognition code predicts that two C2H2-ZFs bind to different DNA triplets, they typically also yield highly dissimilar binding preferences in experimental assays, including B1H assays that are performed on Egr1-fusion constructs and various in vitro and in vivo assays performed on full-length natural C2H2-ZF proteins (Additional file 1: Figure S8b). Thus, there is a low probability that we are erroneously predicting dissimilar sequence preferences, when the preferences are in fact similar. Second, the correlation between pedigree diversity and non-base-contacting DBF score is still significant if we retain only C2H2-ZFs with both: (1) canonical residues at the interface of neighboring C2H2-ZFs, which are least likely to display context effects [7]; and (2) high predicted preference for a specific triplet (greater than twofold) (Additional file 1: Figure S8c). This result argues that context or ambiguous predictions do not dominate the observed trends. Third, and critically, the correlation is highly significant even if we define the pedigree diversity directly based on the combinations of base-contacting residues, rather than based on predicted DNA triplets (Additional file 1: Figure S8d), i.e. pedigrees with high-DBF-score non-base-contacting residues contain more diverse base-contacting residue combinations, regardless of the predicted motifs.

Altogether, we conclude that robustness in DNA binding provided by non-base-contacting residues appears to be a critical factor in diversity of base-contacting residues and, subsequently, the DNA binding preferences of C2H2-ZFs.

### Kaleidoscopic evolution of C2H2-ZF motifs

In a final analysis, we examined more closely the patterns of evolution of the sequence specificity of C2H2-ZF proteins. Specifically, we reasoned that if non-base-contacting residues confer robustness to evolution of base-contacting residues, then the motifs of C2H2-ZFs that possess high-DBF non-base-contacting residues should, on average, evolve more quickly, in addition to simply being more diverse (as assessed above).

**Fig. 7:**
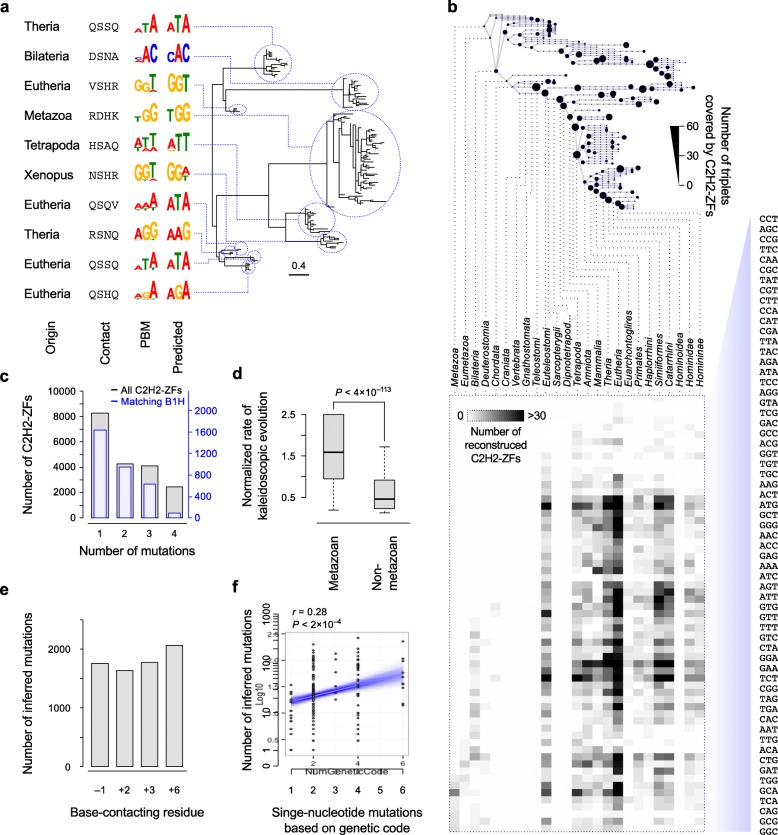
Sequence reconstruction reveals the role of high-DBF non-base-contacting residues in kaleidoscopic evolution of metazoan C2H2-ZFS. **a** Examples of reconstructed ancestral C2H2-ZFs and validation of their motifs by PBM. Only the reconstructed base-contacting residues are shown. The C2H2-ZF pedigrees that are descended from these ancestral C2H2-ZFs are shown on the *right*. **b** The *top panel* shows the number of triplets that were recognized by at least one C2H2-ZF in ancestral organisms, with the number of C2H2-ZFs that recognized each triplet, based on reconstructed sequences, shown in the *bottom panel*. **c** *Histogram* of the number of differences in the base-contacting residues of extant C2H2-ZFs and their most immediate ancestors with a different predicted motif (motif Pearson similarity < 0.1). The *blue bars* include only C2H2-ZFs for which recognition code predictions and B1H motif match (similar to Fig. 2b). **d** Normalized rate of kaleidoscopic evolution (estimated as the ratio of motif-changing mutation rate in base-contacting residues vs. mutation rate at the rest of the C2H2-ZF sequence) for metazoan C2H2-ZFs vs. non-metazoan C2H2-ZFs. **e** Distribution of inferred amino acid transitions between extant metazoan C2H2-ZFs and their most immediate ancestors, across the four base-contacting residues. **f** Each *dot* represents a possible amino acid transition at one of the base-contacting residues, with the *x-axis* showing the number of possible single-nucleotide mutations that can result in that transition and the *y-axis* showing the number of extant-ancestor C2H2-ZF pairs in metazoans that contain that transition

To examine this possibility, we first reconstructed the amino acid sequences of the progenitors of extant C2H2-ZFs by maximum parsimony, allowing us to directly infer the DNA triplets that they recognized from their reconstructed base-contacting residues (see “Methods” for details) [16]. Experimental examination of ten randomly selected reconstructed ancestral C2H2-ZFs using PBMs indicates that the inferred motifs are highly similar to experimental binding preferences (Fig. 7a).

Remarkably, these analyses indicate that the common progenitor of eutherians encoded C2H2-ZFs that, collectively, could bind to nearly all triplets, just as modern mammals do (Fig. 7b). Common ancestors of tetrapods, bony vertebrates (euteleostomes), and even the bilateria also encoded reconstructed C2H2-ZFs binding to many triplets, suggesting that diversity in C2H2-ZF binding preferences was prevalent among early metazoans. Moreover, conservation in sequence preference appears to be the exception rather than the rule in large pedigree expansions: among the 356 extant C2H2-ZFs with a traceable progenitor in the common ancestor of eutherians and pedigree size > 20, only a minority (~37%) bind to the same triplet as their eutherian progenitor. In contrast, in small pedigrees that date to the common ancestor of eutherians (pedigree size ≤ 20), ~86% of extant C2H2-ZFs recognize the same triplet as their eutherian ancestor (3462/4044 extant C2H2-ZFs). This observation suggests that even when all triplets are recognized by the C2H2-ZF repertoire of the genome, the sequence specificity of individual C2H2-ZFs continues to evolve, particularly in the case of large family expansions.

The tree of reconstructed C2H2-ZFs also revealed specifically how mutations have changed the DNA sequence preference of C2H2-ZFs. For each extant C2H2-ZF, we identified the most immediate reconstructed ancestor that recognized a different DNA triplet, which sets an upper limit for the age of the most recent motif change in its lineage. A large fraction (~43%) of these ancestral C2H2-ZFs differ at only one base-contacting residue with their extant descendants (Fig. 7c), indicating that new motifs most often evolve as a result of a single amino acid change in the C2H2-ZF sequence, as previously hypothesized [9, 10], and at a higher rate in metazoans compared to non-metazoans (Fig. 7d). These single amino acid transitions are distributed uniformly across all four base-contacting residues (Fig. 7e), often require only a single nucleotide mutation based on the genetic code (85% of cases), and encompass all possible single-nucleotide missense mutations in metazoans (Fig. 7f), suggesting that such changes are virtually unrestricted.

**Fig. 8:**
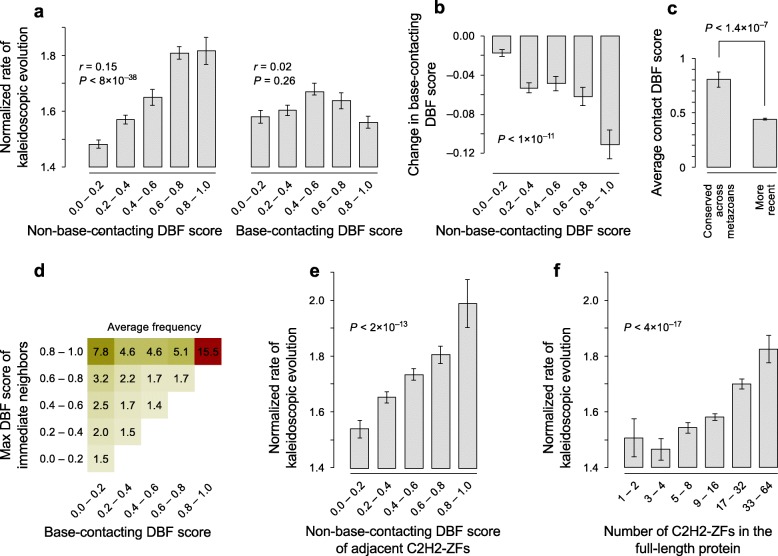
Correlation of kaleidoscopic evolution with DNA-binding affinity of non-base-contacting residues. **a** *Bar graphs* showing evolutionary rates for C2H2-ZFs with varying DBF scores (*left*, non-base contacting; *right*, base-contacting). Extant C2H2-ZFs that have a single amino acid transition at the base-contacting residues and a different motif relative to their inferred ancestor (Pearson similarity < 0.1) are included. The normalized rate of kaleidoscopic evolution is defined as the ratio of mutation rate in base-contacting residues relative to the rest of the C2H2-ZF sequence. *P* values are based on Fisher’s transformation of Pearson correlation between rate of kaleidoscopic evolution and DBF score. **b** Change in DBF score of base-contacting residues of extant C2H2-ZFs relative to their ancestors, with bins corresponding to DNA-binding strength of non-base-contacting residues. *P* value calculations are the same as in (**a**). **c** Comparison of DBF scores for ancient vs. recent metazoan C2H2-ZFs. The first bin corresponds to C2H2-ZFs from high-confidence pedigrees with conserved base-contacting residues that originated in the last common ancestor of metazoans. The second bin corresponds to C2H2-ZFs from high-confidence pedigrees with base-contacting residues that date back to the ancestor of chordates or more recent. *Error bars* represent the standard error. The *P* value is based on Student’s t-test. **d** Average frequency of base-contacting residue combinations, plotted in bins of DBF score of base-contacting residue combinations themselves (*x-axis*), and maximum DBF score of their first-degree neighbors, i.e. other combinations that are only different at a single residue (*y-axis*). For example, a base-contacting residue combination with a DBF score of 0–0.2 that can be generated by a single mutation from another combination with a DBF score of 0.8–1 is found, on average, in 7.8 C2H2-ZFs. **e** The rate of kaleidoscopic evolution for C2H2-ZFs neighboring C2H2-ZFs of varying DBF score or (**f**) in varying C2H2-ZF array length. *P* values are based on Fisher’s transformation of Pearson correlations

As predicted by our model, the rate of evolution in metazoan C2H2-ZFs has a striking correlation with the DBF score of the non-base-contacting residues, but not the base-contacting residues (Fig. 8a and Additional file 1: Figure S9a, b), further supporting the notion that high-DBF non-base-contacting residues facilitate evolution of sequence specificity. Moreover, in recent C2H2-ZF lineages, the DBF scores for base-contacting residues have generally decreased relative to the inferred ancestral protein. The rate of this reduction is significantly higher in C2H2-ZFs with high DBF scores for non-base-contacting residues (Fig. 8b and Additional file 1: Figure S9c, d), further supporting relaxed constraint on base-contacting residues as a contributor to larger motif diversity. In contrast, C2H2-ZFs with highly conserved recognition motifs whose origin dates to the base of metazoans tend to have base-contacting residue combinations with very high DBF (Fig. 8c), suggesting that ancient metazoan C2H2-ZFs were more similar to non-metazoan C2H2-ZFs in this regard and that extant low-DBF base-contacting residues have evolved by successive mutations of ancient high-DBF base-contacting residue combinations. Indeed, there is a clear correlation between the global frequencies of low-DBF base-contacting residues in metazoans and how easily these residue combinations can be created from high-DBF combinations by mutation (Fig. 8d).

## Discussion and Conclusions

Our analyses indicate that changes in DNA-binding preference as a result of small mutations in DNA-binding domain is pervasive among metazoan C2H2-ZFs evolutionary time and has shaped both the diversity of binding motifs and the frequency landscape of base-contacting residue combinations among modern C2H2-ZFs. These changes are facilitated in metazoans by contributions of non-base-contacting residues to hydrogen bonding between DNA and protein. To our knowledge, the extent of this phenomenon and its mechanistic underpinning have not been previously described. To describe these evolutionary patterns in the evolution of C2H2-ZFs, we introduce the term “kaleidoscopic,” because the patterns are analogous to a kaleidoscope in several ways. First, slight changes in C2H2-ZF residues can cause dramatic changes in the sequences to which they bind. Second, the combinations of specificity residues and potential motifs appear virtually unrestricted. Third, the evolutionary origins of any given C2H2-ZF are difficult to trace, because they can arise by multiple means. Kaleidoscopic evolution of C2H2-ZFs is ancient, ongoing, and widespread, and may be even more prevalent than our estimate in Fig. 7c, since we are often not able to reconstruct the sequences of intermediate ancestors that may recognize a different binding site, despite having fewer amino acid differences relative to their extant descendants.

The observation that kaleidoscopic evolution of new binding preferences in C2H2-ZF proteins occurs in addition to duplication, rearrangement, and shuffling of existing C2H2-ZFs is consistent with the known prevalence of positive selection on base-contacting residues in various metazoans [9, 10] and with studies directly showing the contribution of recent mutations to recognition of new binding sites by KRAB-C2H2-ZF proteins [15]. Given the extremely large number of C2H2-ZF proteins in metazoan, and particularly eutherian, genomes, it is conceivable that the maintenance of a full collection of DNA triplet preferences is not due to pressure that all are present, but rather to the statistical improbability that none are missing, considering the ease of their appearance by kaleidoscopic evolution.

Mechanistically, our model indicates that the functional robustness of DNA binding conferred by non-DNA-contacting residues in metaozans has been a critical factor for the plasticity of the C2H2-ZF binding vocabulary. Nonetheless, other factors may have also contributed. For example, interaction of each C2H2-ZF with DNA could be supported not only by non-base-contacting residues of the same C2H2-ZF, but also by strong binding of neighboring zinc fingers in the context of multi-ZF arrays. Indeed, we observed that in metazoans, rapidly evolving C2H2-ZFs are often accompanied by neighboring C2H2-ZFs with high-DBF non-base-contacting residues (Fig. 8e), suggesting that high-DBF C2H2-ZFs confer mutational robustness to their adjacent C2H2-ZFs through stabilization of protein-DNA interaction. In addition, C2H2-ZFs in longer arrays evolve more rapidly (Fig. 8f), further supporting the role of cooperative binding of C2H2-ZFs in diversification of their binding sites.

Finally, we note that our proposed mechanism for diversification of C2H2-ZFs through mutational robustness is analogous to previous observations in enzymes, where protein structures that are robust to mutations have been found to represent greater enzymatic diversity [28]. Therefore, this concept readily extends beyond the evolution of C2H2-ZFs and functional robustness might represent a general prerequisite for evolution of diversity in many protein-ligand interactions.

## Methods

### Prediction of triplets for eukaryotic C2H2-ZFs

We obtained the protein sequences of predicted TFs for 283 sequenced eukaryotic genomes from CisBP [29] and scanned them for matches to the Pfam model of C2H2-ZF domain (PF00096). Redundant C2H2-ZFs (with identical sequences) were removed and those with a length of 23 AA, corresponding to the most abundant form of C2H2-ZFs, were selected, resulting in a collection of 106,771 unique C2H2-ZFs. For each C2H2-ZF, the three-nucleotide binding preference was predicted from base-contacting residues using a previously published recognition code [16] and the most preferred DNA triplet for each C2H2-ZF was identified.

### Protein-binding microarray (PBM) analysis

PBM methods, including quality control and data analysis, followed the procedure described previously [29, 30]. Briefly, we polymerase chain reaction (PCR)-amplified, sub-cloned, and sequence-verified inserts encompassing 43 full-length non-metazoan C2H2-ZF proteins, as well as ten reconstructed ancestral C2H2-ZF domains in the F3 context of mouse Egr1, and followed the previously described PBM laboratory methods using two arrays with different probe sequences [30]. For each array, we calculated 8-mer Z-scores as previously described [31]. For full-length proteins, the best-performing motif from four different algorithms [29, 32] based on cross-replicate array evaluation was selected and compared to the motif that was predicted from protein sequence using a previously described motif alignment algorithm [16]. For reconstructed ancestral C2H2-ZF domains in the Egr1 context, we used linear regression, with 8-mer sequences that match the NNNNGGGC pattern as the covariates and the PBM Z-score as the response variable, as described previously [16]. The PBM data are available at GEO (http://www.ncbi.nlm.nih.gov/geo/) under accession number GSE73117.

### De-convoluting the DBF model

To identify the contribution of different C2H2-ZF residues to DNA binding, we started from a previously described DBF model [16], which uses the identity of the 12 residues between the zinc-binding cysteine and histidine residues to predict whether a C2H2-ZF can bind to any DNA sequence, based on a random forest classifier that outputs a binding probability score between 0 and 1. We de-convoluted this model into two random forest regression models, each measuring the impact of either the base-contacting or non-base-contacting residues on DBF. To do so, we used the original DBF model to score 1,000,000 random AA sequences of length 12. We then used these 1,000,000 scores as the response variable to train the base-contacting DBF model using only the four residues that corresponded to base-contacting C2H2-ZF positions as covariates and also to train the non-base-contacting DBF sub-model using only the other eight residues that corresponded to non-base-contacting C2H2-ZF positions. Each of the two sub-models is a random forest predictor that outputs a score between 0 and 1.

### MD simulations

The base-contacting residues of 146 natural C2H2-ZFs, whose binding preference was previously determined by PBMs in the F3 context of mouse Egr1 [16], were randomly swapped and both the original and recombinant C2H2-ZFs were modeled in complex with their respective optimal binding sites. Models were generated using the PDB file 1ZAA [33] for a template with the crystallographic water molecules and counter-ions removed. The Jackal [34] program was used to model the protein, while the DNA component was mutated using Chimera [35] to produce the requisite sequence. The DNA was elongated by 4 bp at each end using X3DNA [36], such that any end effects (termini melting) would not affect the protein-bound nucleotides. Zinc 2+ ions were approximated using four mass-less dummy atoms in addition to the core zinc, each with a 1/2 positive charge, in a tetrahedral arrangement, for correctly orientated coordination of the four C2H2-ZF side-chains [37]. A total of 292 models were constructed, comprising 146 original and 146 recombinant domains. All models were prepared for Amber 10 [38], using the WHATIF web interface [39] to build in any missing atoms and identify protonation states. Each model was energy-minimized using a combination of steepest descent and conjugate gradient methodology. All known Lys and Arg rotamers were identified from the Dunbrack library [40] and in each case the rotamer with closest proximity to a DNA-PO4 group was identified. Hydrogen bonds and salt bridge distances and angles were detected between C2H2-ZFs and DNA using ptraj. The frequency of each with respect to either sugar phosphate backbone interaction or nitrogenous base interaction was output for each model.

### Construction of an evolutionary tree of eukaryotic C2H2-ZFs and identification of high-confidence pedigrees

We constructed a maximum likelihood tree of 106,771 unique C2H2-ZFs that have the canonical length of the Pfam model PF00096 (23 AA), taken from protein annotations of 283 sequenced genomes [29]. After masking the base-contacting residues (by replacing them with gaps), a maximum likelihood tree was constructed from the sequences using FastTree [41], with branch lengths optimized under assumption of variable rates of evolution over sites [42].

For identification of the origin of families (i.e. sub-trees) within the tree of C2H2-ZFs, we first assigned the origin of each family to the last common ancestor (LCA) of all the species that had, in their genome, at least one C2H2-ZF of that family. The LCA was determined based on the tree of life, obtained from the NCBI taxonomy database [27]. Next, we asked whether this assignment was compatible with a maximum parsimony explanation of the emergence and loss of that family among the species. To do so, we identified, on the tree of life, all the extant species that had at least one C2H2-ZF of the family of interest, and labeled those species with “1” and all other species with “0.” Then we assigned 1 s and zeros to the internal nodes of the tree of life in order to minimize the number of transitions from 1 s to zeros or from zeros to 1 s along the edges of the tree. If the above-mentioned LCA was labeled “1,” and no other node represented a transition from zero to 1, then we considered that LCA to be compatible with a maximum parsimony explanation of the history of the C2H2-ZF family. Consequently, that family was considered to be a high-confidence “pedigree.” The maximum parsimony assignment of 1 s and zeros was found using Fitch’s algorithm [43].

### Measuring recognition diversity in pedigrees

To measure the diversity of DNA triplets recognized by extant C2H2-ZFs of a pedigree, we first calculated the “effective number of triplets” (ENT) for each pedigree as the harmonic mean of the square of relative triplet frequencies in that pedigree, modeled after the previously described “effective number of codons” [44]. *ENT = 1/Σ*_*i*_ *f*_*i*_^*2*^, where *f*_*i*_ is the relative frequency of C2H2-ZFs within the pedigree that recognize DNA triplet *i*. The recognition diversity (RD) in a pedigree is calculated as the ratio of the ENT in that pedigree to the maximum obtainable ENT, which is equal to the pedigree size or 64, whichever is smaller: *RD = ENT/ENT*_*max*_.

### Reconstruction of ancestral C2H2-ZF sequences

We used maximum parsimony to infer ancestral sequences from the tree of C2H2-ZFs. Each of the 23 positions in the sequence of each ancestral node was assigned to one of the 20 amino acids, so that the number of changes along the edges of the tree was minimized. This operation was performed for each position separately, using Fitch’s algorithm [43]. For prediction of ancestral motifs using the C2H2-ZF recognition code [16], only those ancestral sequences were considered for which all of the base-contacting residues could be inferred unambiguously, (i.e. only a single amino acid could be assigned to each residue so as to meet the maximum parsimony criteria).

### Measuring the rate of kaleidoscopic evolution

Using the predicted DNA triplets for the reconstructed ancestral sequences, for each extant C2H2-ZF, we identified the most immediate ancestor that recognized a different DNA motif (Pearson correlation < 0.1), limiting our search to ancestral nodes that represented the progenitor of a high-confidence pedigree. We further filtered these extant/ancestral C2H2-ZF pairs to retain only those that differed at only one of the four base-contacting residues, representing recent single mutations that have changed the recognition motif of the C2H2-ZF. For these lineages, the average number of mutations per base-contacting site is therefore 1/4 = 0.25, whereas the average per-site mutation rate for the rest of the C2H2-ZF sequence is equal to the sum of the lengths of the branches that connect the ancestral sequence to the extant C2H2-ZF, calculated by FastTree [41]. We calculated the normalized rate of kaleidoscopic evolution as the ratio of mutation rate in base-contacting residues (0.25) to the rest of the C2H2-ZF sequence, which essentially measures the excess or dearth of mutations at base-contacting sites relative to the rest of the C2H2-ZF sequence.

## Additional files

Additional file 1: Supplementary Figures. **Figure S1.** Overview of C2H2-ZF prevalence in metazoans. **Figure S2.** Number of C2H2-ZFs with highest preference for each DNA triplet. **Figure S3.** Multiple C2H2-ZFs recognize each triplet in metazoans. **Figure S4.** C2H2-ZFs with related contact residue combinations recognize the same triplets in metazoans and non-metazoans. **Figure S5.** Evaluation of DBF model. **Figure S6.** Correlation between non-base-contacting DBF score and the salt bridge formation arising from Lys and Arg rotamer proximity to DNA PO4 groups. **Figure S7.** Origins of C2H2-ZF pedigrees. **Figure S8.** Robustness of diversity analysis to inaccuracies in the recognition code. **Figure S9.** Correlation of kaleidoscopic evolution with DNA-binding affinity of non-base-contacting residues. (PDF 3378 kb)

Additional file 2: Table S1. Inferred divergence time of C2H2-ZFs for pairs of human-mouse orthologs. (XLSX 372 kb)
