# Supplementary material for: Non-base-contacting residues enable kaleidoscopic evolution of metazoan C2H2 zinc finger DNA binding
Source: Genome Biol. 2017 Sep 6;18:167. doi: 10.1186/s13059-017-1287-y (PMC5588721; doi:10.1186/s13059-017-1287-y)
Supplement: Supplementary file 1 — Supplementary Figures. Figure S1. Overview of C2H2-ZF prevalence in metazoans. Figure S2. Number of C2H2-ZFs with highest preference for each DNA triplet. Figure S3. Multiple C2H2-ZFs recognize each triplet in metazoans. Figure S4. C2H2-ZFs with related contact residue combinations recognize the same triplets in metazoans and non-metazoans. Figure S5. Evaluation of DBF model. Figure S6. Correlation between non-base-contacting DBF score and the salt bridge formation arising from Lys and Arg rotamer proximity to DNA PO4 groups. Figure S7. Origins of C2H2-ZF pedigrees. Figure S8. Robustness of diversity analysis to inaccuracies in the recognition code. Figure S9. Correlation of kaleidoscopic evolution with DNA-binding affinity of non-base-contacting residues. (PDF 3378 kb) [file 13059_2017_1287_MOESM1_ESM.pdf]

Supplementary Figures for

Non-base-contacting residues enable kaleidoscopic evolution of metazoan C2H2 zinc finger DNA binding

Hamed S. Najafabadi[1,2,3,*], Michael Garton[3], Matthew T. Weirauch[4,5], Sanie Mnaimneh[3], Ally Yang[3], Philip M. Kim[3,6,7], Timothy R. Hughes[3,5,7,*]

[1] Department of Human Genetics, McGill University, Montreal, QC, Canada

[2] McGill University and Genome Quebec Innovation Centre, Montreal, QC, Canada

[3] Donnelly Centre for Cellular and Biomolecular Research, University of Toronto, Toronto, Canada

[4] Center for Autoimmune Genomics and Etiology, and Divisions of Biomedical Informatics and Developmental Biology, Cincinnati Children's Hospital Medical Center, Cincinnati, Ohio, USA.

[5] Canadian Institute for Advanced Research, Toronto, Ontario, Canada.

[6] Department of Computer Science, University of Toronto, Toronto, Canada

[7] Department of Molecular Genetics, University of Toronto, Toronto, Canada

* Correspondence should be addressed to H.S.N. (hamed.najafabadi@mcgill.ca) or T.R.H. (t.hughes@utoronto.ca).

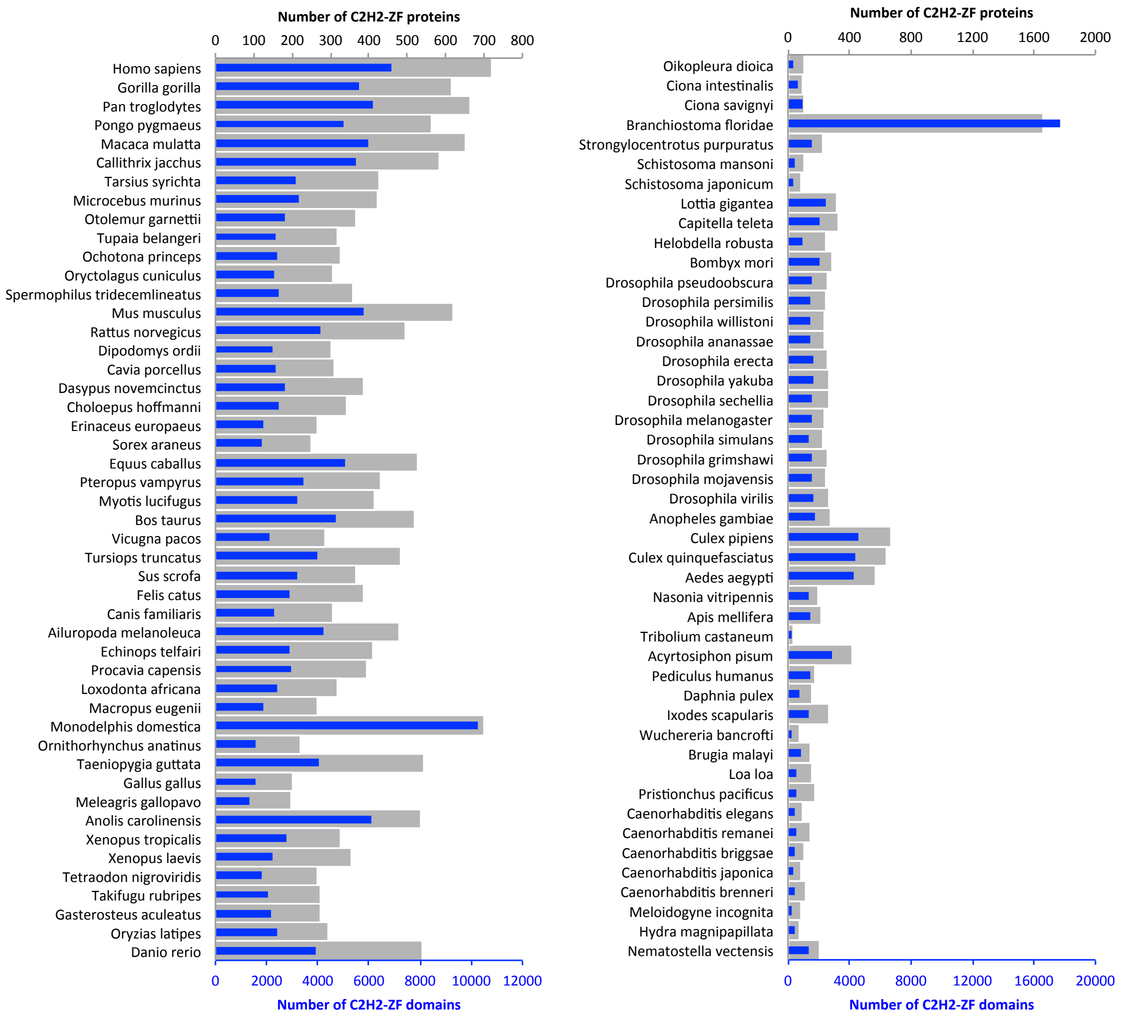

Figure S1. Overview of C2H2-ZF prevalence in metazoans. Number of C2H2-ZF proteins (grey, upper axis) as well as individual C2H2-ZF domains (blue, lower axis) in metazoan genomes. Organisms are sorted by their phylogenetic relationship similar to Figure 2. Vertebrates are shown on the left. C2H2-ZF numbers are obtained by scanning C2H2-ZF protein sequences from CisBP[1] using Pfam model PF00096.

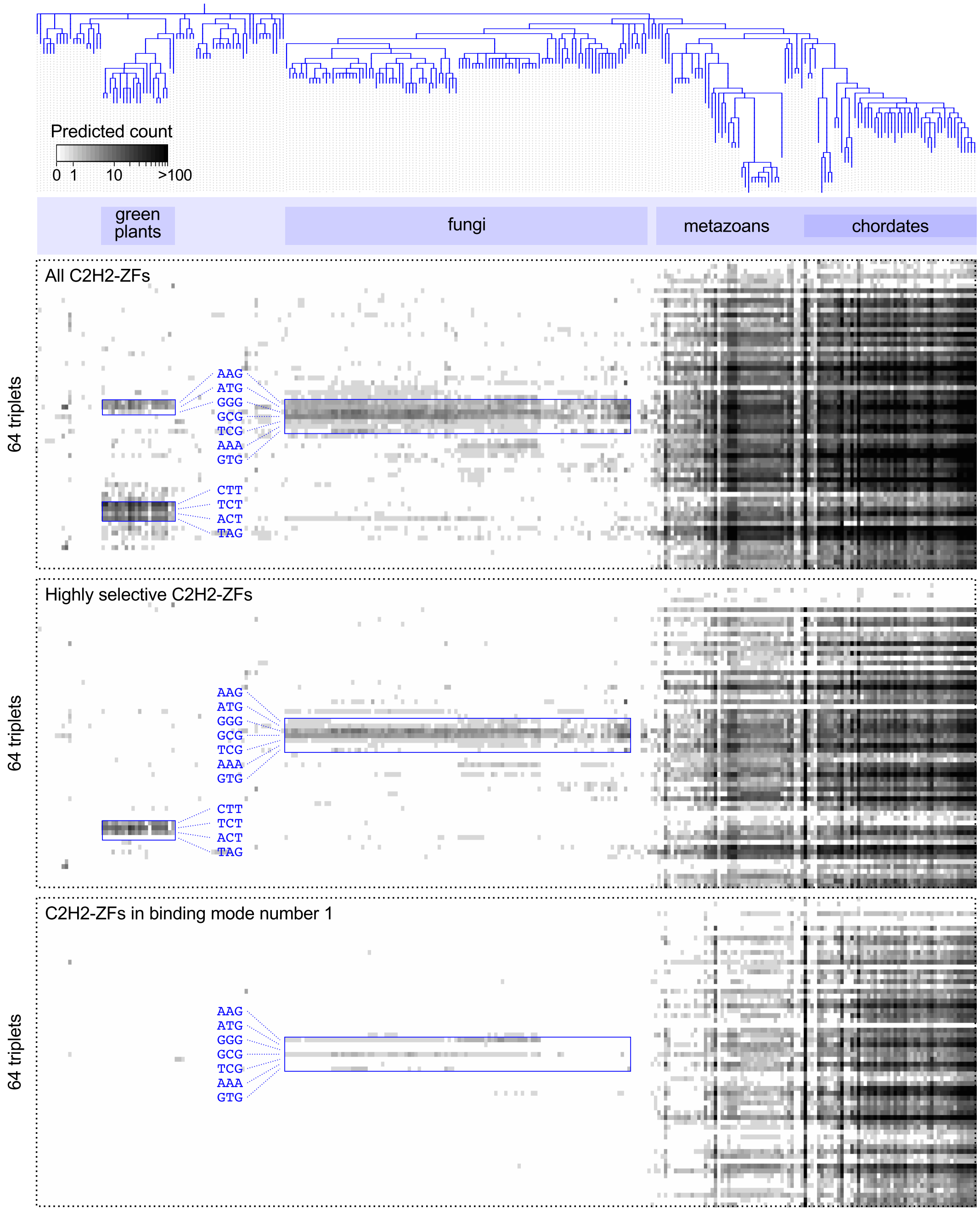

Figure S2. Number of C2H2-ZFs with highest preference for each DNA triplet. The order of triplet (y-axis) and organisms (x-axis) is the same as **Figure 1a**. In the three heatmaps, in order from top to bottom, the following C2H2-ZFs are included: (**Top**) All C2H2-ZFs with a canonical length of 23 (same as in **Figure 2**); (**Middle**) C2H2-ZFs of length 23, whose predicted motifs are highly selective, meaning that the most preferred DNA triplet is at least twice as likely to be bound than the second most preferred DNA triplet; (**Bottom**) C2H2-ZFs of length 23, whose binding context in the multi-ZF arrays matches the canonical "binding mode #1" according to Garton et al.[2]. Binding modes represent the variations in the orientation of the ZF relative to DNA, and are mainly defined based on boundary residues at positions -2 of the ZF of interest and position +9 of its preceding ZF in the multi-ZF array. Binding mode #1 was selected here because the recognition code that was used for predicting DNA triplets was trained on B1H data in the context of mouse Egr1-ZF3, which also uses binding mode #1.

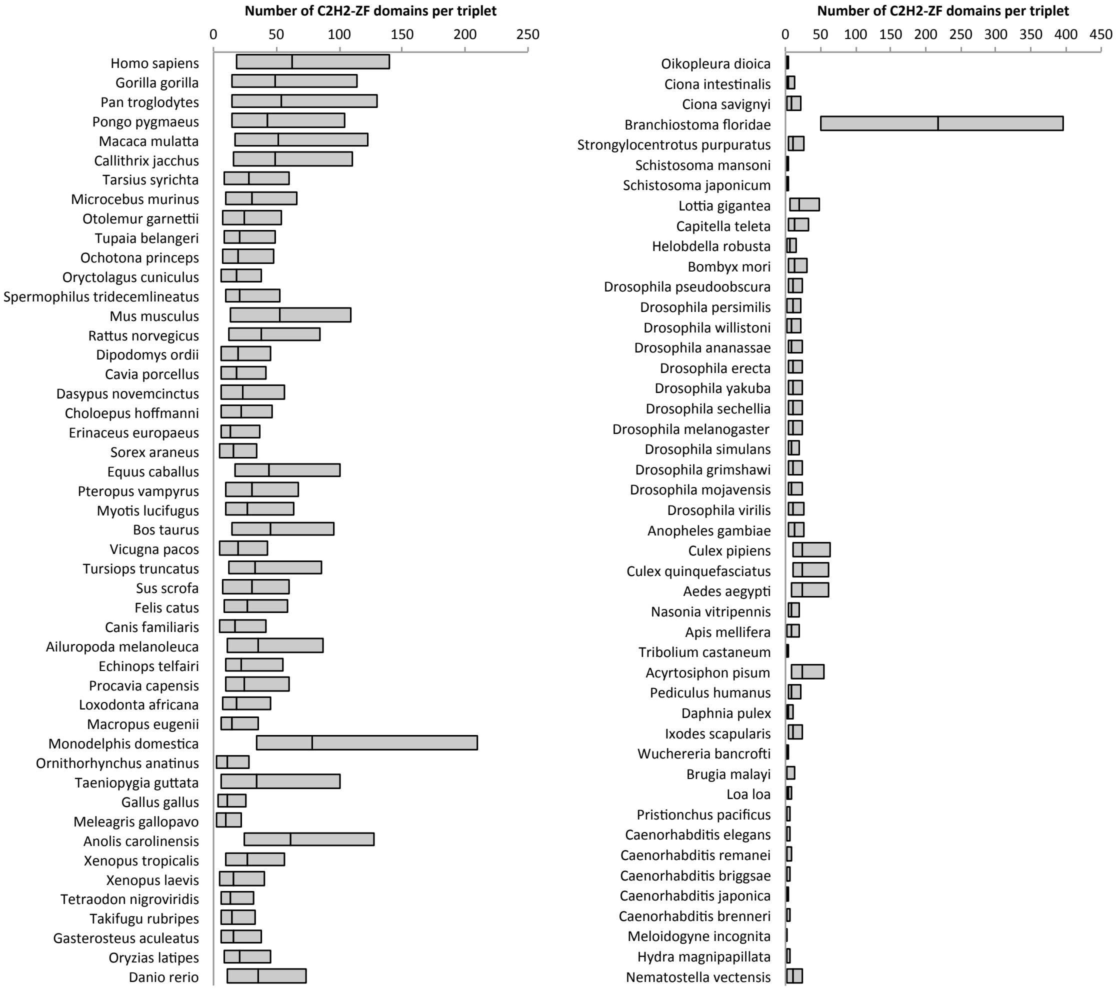

Figure S3. Multiple C2H2-ZFs recognize each triplet in metazoans. The bar plots represent the lower and upper quartile of the number of C2H2-ZFs that are predicted to have highest preference for each triplet, with the median shown using the centroid line. Vertebrates are shown on the left.

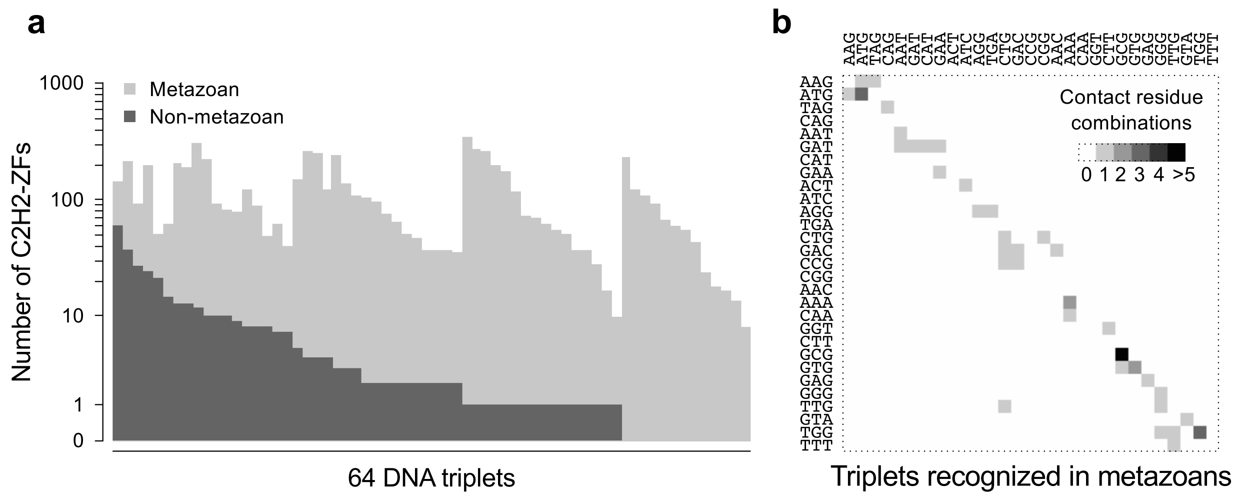

Figure S4. C2H2-ZFs with related contact residue combinations recognize the same triplets in metazoans and non-metazoans. (**a**) Number of C2H2-ZFs with highest preference for each DNA triplet, based on experimental motifs obtained by B1H for 7006 metazoan C2H2-ZFs and 366 non-metazoan C2H2-ZFs[3]. (**b**) Triplets most preferred by 48 unique base-contacting residue combinations that were present in at least one metazoan and one non-metazoan C2H2-ZF, in previously reported B1H data[3]. For each base-contacting residue combination, the average metazoan motif and the average non-metazoan motif were calculated, and the triplet with the maximum motif score was assigned to each combination in each lineage. Each element of the heat map shows the number of combinations that recognize the triplet on the left in non-metazoans, and the triplet on the top in metazoans. The rows and columns are in the same order, such that the diagonal represents recognition of the same triplet in both lineages.

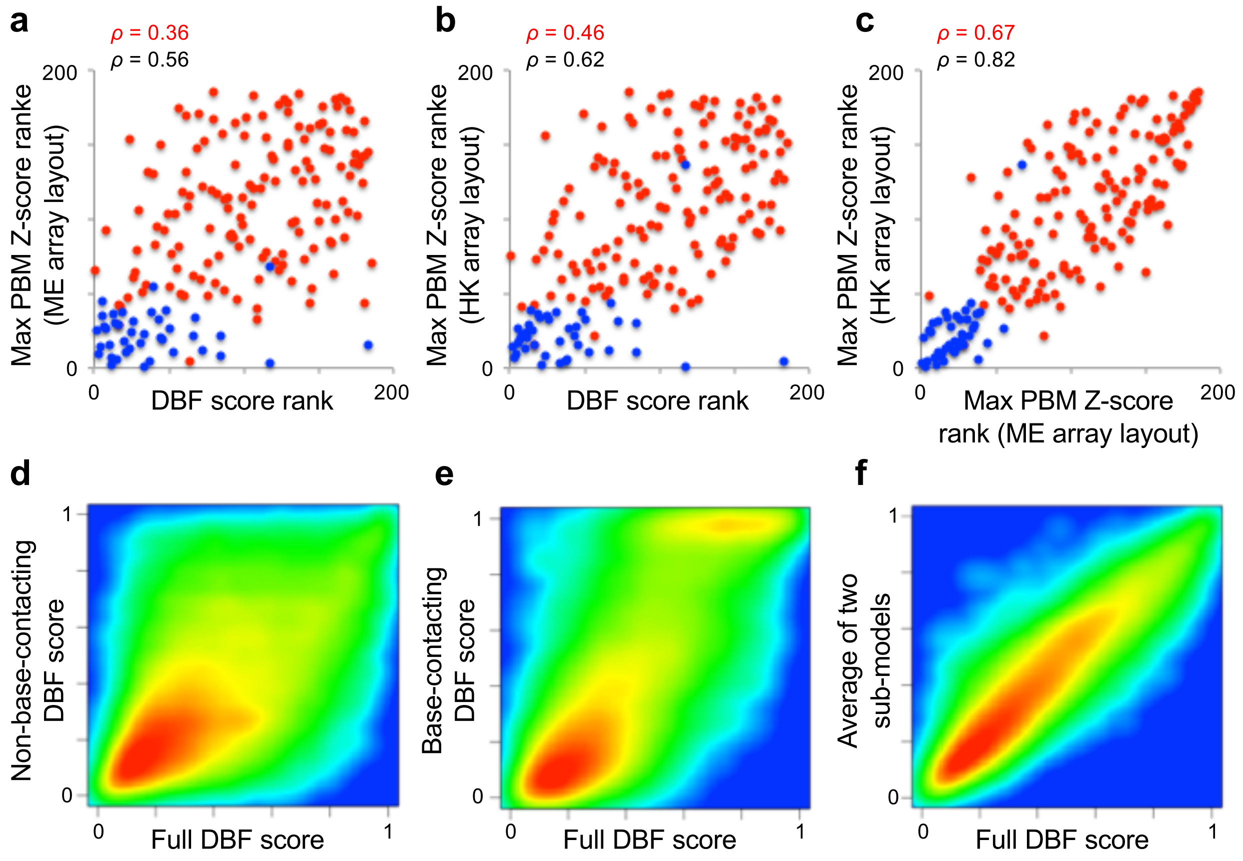

Figure S5. Evaluation of DBF model. (**a-c**) Correlation between DBF score and maximum PBM Z-score. Axes show the ranks of 185 C2H2-ZFs with respect to their DBF scores or previously reported maximum PBM Z-scores[3]. The red and blue dots correspond to PBMs that are labeled as successful or failed, respectively. The Spearman correlations for successful PBMs and all PBMs are denoted on top of each panel in red and black, respectively. The scatterplots of DBF vs. PBM Z-score for each of ME and HK array layouts are shown in (**a**) and (**b**), respectively. The ME and HK array Z-scores are directly compared in the (**c**), providing an upper bound on the obtainable correlation. (**d-f**) DBF sub-models for base-contacting and non-base-contacting residues. Two additional random forests were trained using previously published B1H data[3] to classify whether a C2H2-ZF binds any specific DNA sequences, one with only the C2H2-ZF base-contacting residues and the other with only non-base-contacting residues as the classifier covariates. Each contour plot shows the distribution of DBF scores for 106,771 eukaryotic C2H2-ZFs, with red, green and blue representing high-, medium- and low-density regions, respectively. **d, e,** and **f** show the correlation of scores produced by each of the sub-models and their average, respectively, against scores produced by the full model. Note that the linear combination of sub-models in **f** cannot take into account the covariate interactions between base-contacting and non-base-contacting residues.

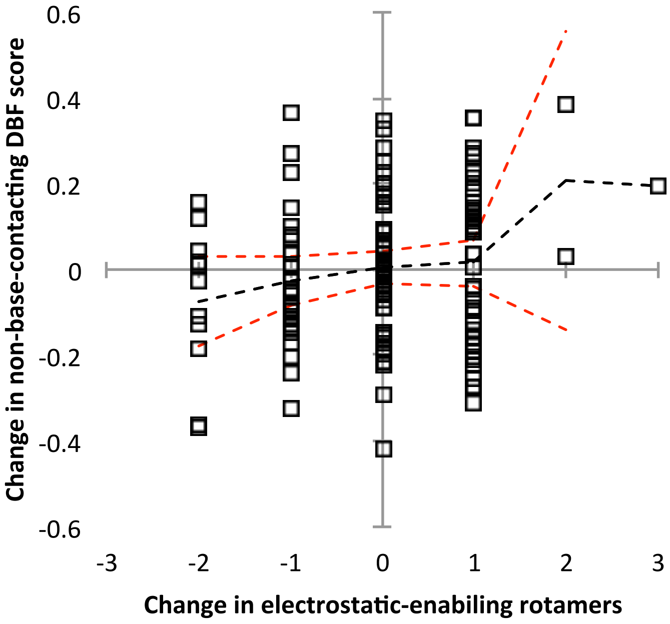

Figure S6. Correlation between non-base-contacting DBF score and the salt bridge formation arising from Lys and Arg rotamer proximity to DNA PO4 groups. The changes in backbone DBF score and electrostatic-enabling rotamers were calculated using the same pairs of natural/recombinant C2H2-ZFs as in **Figure 2b-d**. The black dashed line represents the average DBF score, and the red dashed lines represent the 95% confidence interval for the average. Pearson correlation is 0.19 ($P < 0.01$).

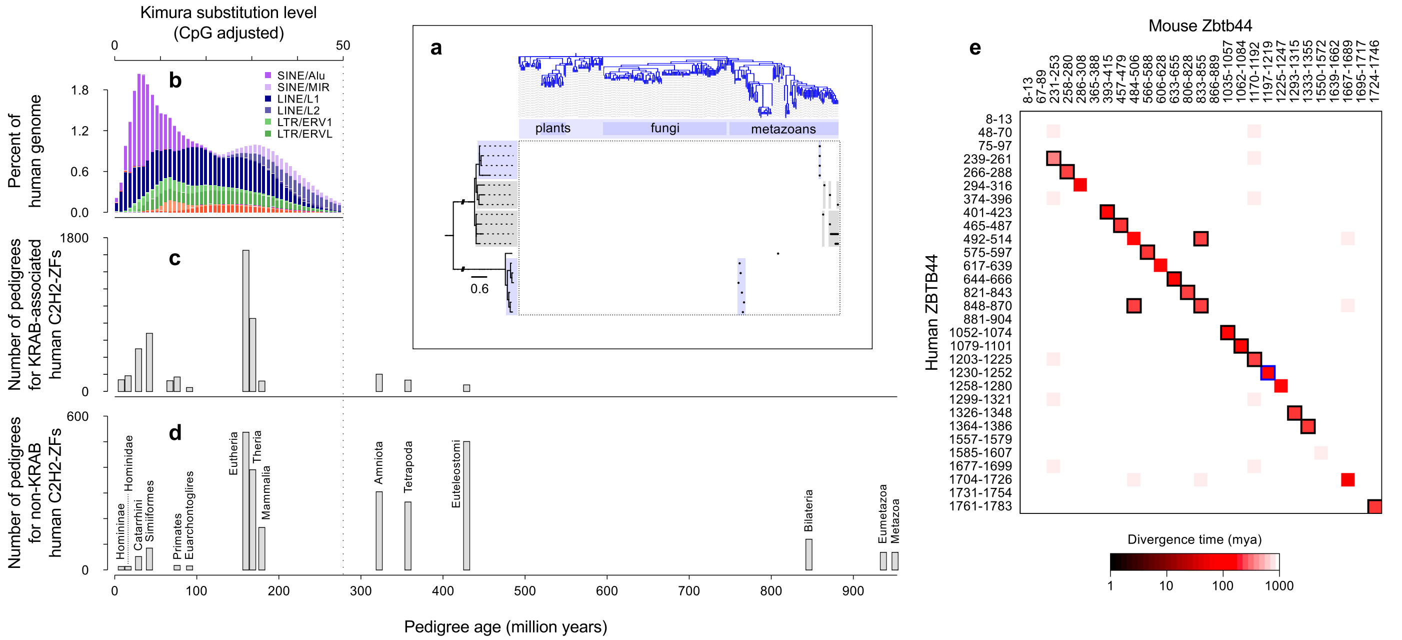

Figure S7. Origins of C2H2-ZF pedigrees. (**a**) Two examples of high-confidence pedigrees are highlighted in blue, and two phylogenetic clusters that are likely missing several extant C2H2-ZFs from other species are highlighted in grey. The phylogram on the left depicts example C2H2-ZFs, the phylogram on the top represents the species tree, and the dots in the matrix mark the organisms that contain at least one copy of each C2H2-ZF. High-confidence pedigrees follow a maximum-parsimony pattern of gain/loss in the species tree, and usually contain all or most of the species from a specific taxon (blue boxes in the matrix). In contrast, C2H2-ZF groups that are missing extant members (due to incomplete genome annotations or erroneous reconstruction of the C2H2-ZF phylogenetic tree) require multiple gain/loss events in the species tree to explain their evolutionary history, which is not compatible with maximum parsimony. The C2H2-ZF phylogenetic tree is reconstructed using the C2H2-ZF sequences excluding the base-contacting residues, which are highlighted in yellow. (**b-d**) The evolutionary history of human KRAB- and non-KRAB-associated C2H2-ZFs within high-confidence pedigrees, and the overlap of origin of KRAB-associated C2H2-ZFs with invasion of retroelements. (**b**) Histogram of the divergence of retroelements relative to their consensus sequence, taken from RepeatMasker[4]. Two major periods of retroelement invasion can be seen as two peaks in the histogram. (**c**) Histogram of the age of human KRAB-associated C2H2-ZF pedigrees. (**d**) Histogram of the age of human non-KRAB C2H2-ZF pedigrees. For determining the age of pedigrees, the time of divergence of the ancestral organism that maps to the origin of each pedigree was used[5]. The x-axis for (**b**) is scaled so that each unit of substitution corresponds to 5.44 million years in (**c**) and (**d**), as previously determined[3]. (**e**) Inferred divergence time of C2H2-ZF pairs for a representative pair of human-mouse orthologs. The numbers represent the start and end of C2H2-ZFs in the full-length protein. Black borders highlight pairs of C2H2-ZFs that belong to a high-confidence pedigree. Blue borders stand for pairs of C2H2-ZFs with identical sequences. See **Additional file 2** for all pairwise comparisons of C2H2-ZF orthologs between human and mouse.

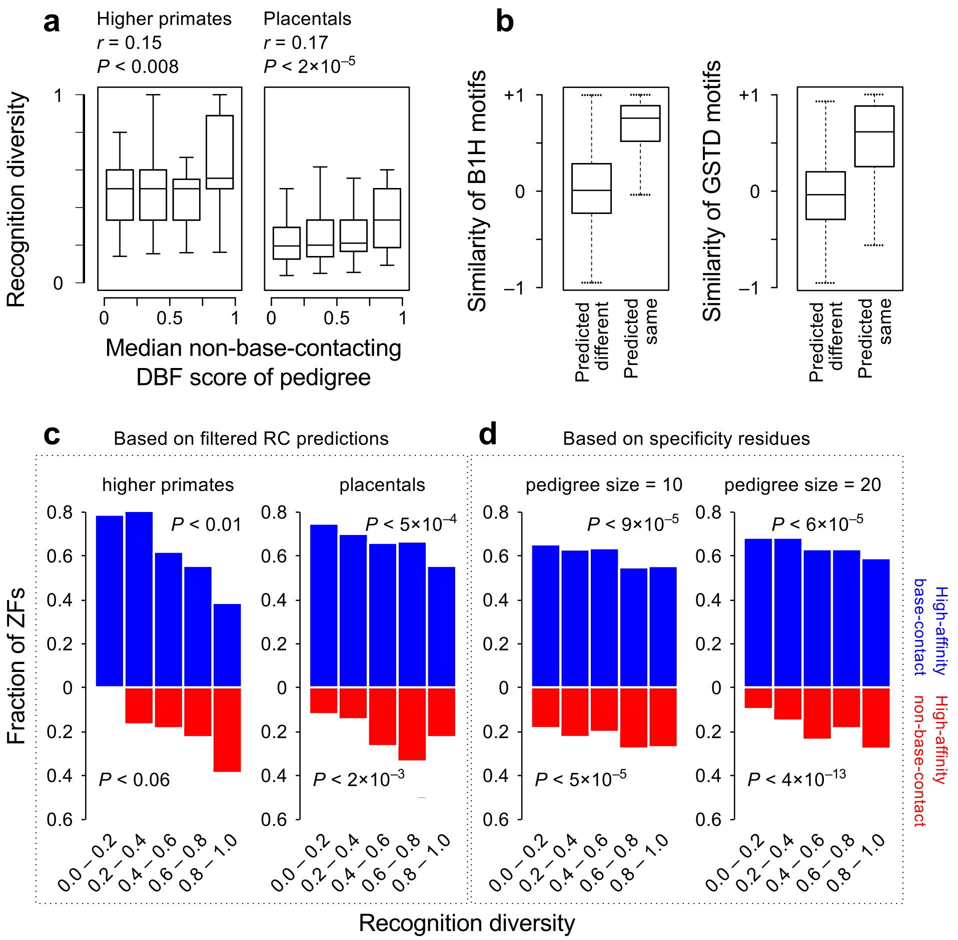

Figure S8. Robustness of diversity analysis to inaccuracies in the recognition code. (**a**) Barplots of recognition diversity vs. median non-base-contacting DBF score of C2H2-ZF pedigrees that originated in the ancestor of higher primates (left) or placentals (right). The Pearson correlation of recognition diversity vs. median non-base-contacting DBF score is shown on top of each graph along with the associated P-value. (**b**) Comparison of the similarity of experimentally measured motifs for pairs of C2H2-ZFs that are predicted to bind to the same or different DNA triplets, based on (left) B1H data obtained from >8000 individual C2H2-ZFs in fusion with F1-F2 region of mouse Egr1[3], or (right) based on motifs obtained from various full-length proteins in PBM, SELEX, or ChIP-seq experiments[3]. (**c**) Fraction of high- and low-affinity non-base-contact residues in pedigrees with varying recognition diversity. Calculations are similar to **Figure 6c**, except that only a subset of high-confidence C2H2-ZF motif predictions are included, consisting of C2H2-ZFs that use the canonical “binding mode” number 1 according to Garton et al.[2], and are highly selective, meaning that the most preferred DNA triplet for each C2H2-ZF is twice as likely to be bound compared to the second most preferred DNA triplet. Binding modes are defined based on boundary residues at positions -2 of the ZF of interest and position +9 of its preceding ZF in the multi-ZF array[2]. (**d**) Same as **Figure 6c**, except pedigree diversity is defined based on the number of different specificity residue combinations rather than the number of different predicted DNA triplets.

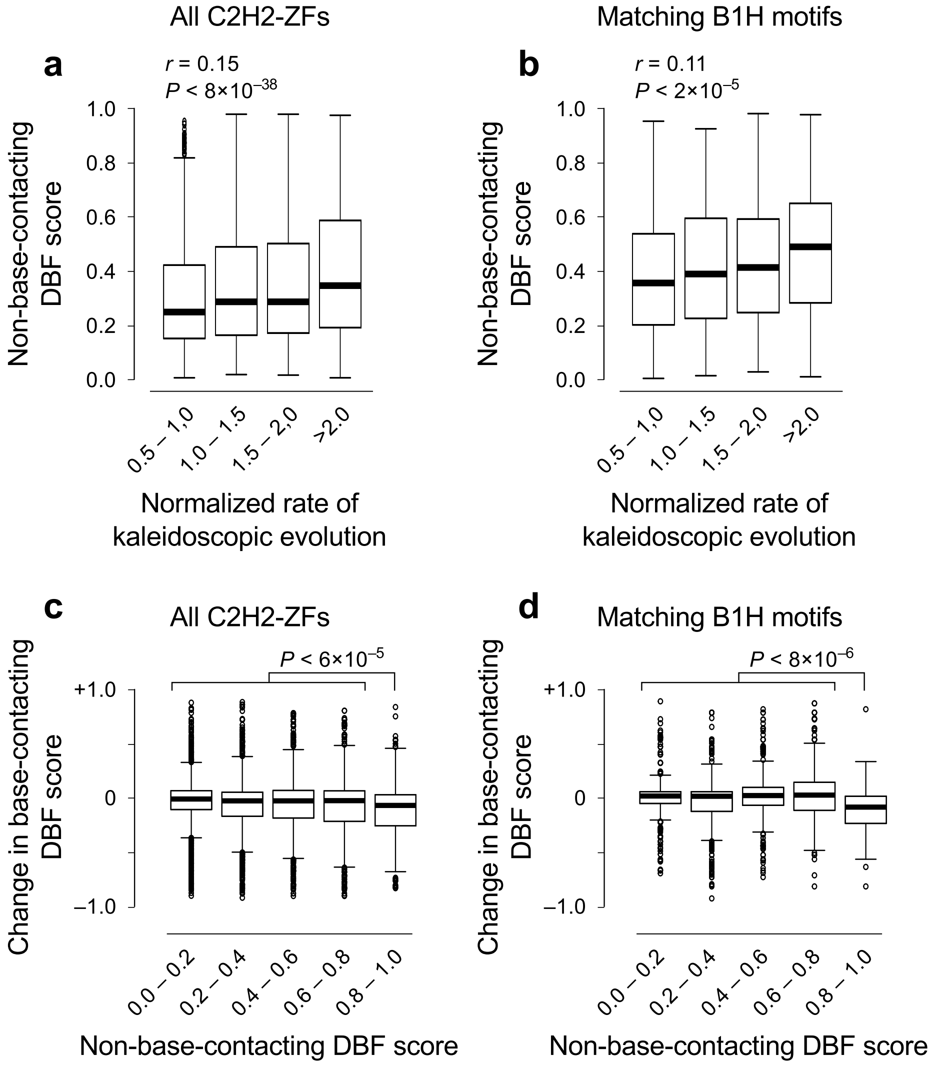

Figure S9. Correlation of kaleidoscopic evolution with DNA-binding affinity of non-base-contacting residues. (**a**,**b**) The box plot in (**a**) shows the distribution of non-base-contacting DBF scores for C2H2-ZFs with varying rates of kaleidoscopic evolution. The box plot in (**b**) includes only C2H2-ZFs with matching predicted and experimental motifs, i.e. C2H2-ZFs whose base-contacting residues match at least one experimentally examined C2H2-ZF[3,6] and whose recognition code-predicted motif matches the experimental motif (Pearson correlation >0.9). The bottom and top of the boxes represent the first and third quartiles. The whiskers represent 1.5 times the interquartile rxange. (**c**,**d**) The box plots represent the distribution of change in base-contacting DBF scores for C2H2-ZFs with different non-base-contacting DBF scores. The box plot in (**c**) corresponds to all extant-ancestor C2H2-ZF pairs, and (**d**) represents pairs of extant and ancestral C2H2-ZFs filtered for high-confidence motif predictions (similar to **b**).

REFERENCES

1 Weirauch, M. T. *et al.* Determination and inference of eukaryotic transcription factor sequence specificity. *Cell* **158**, 1431-1443, doi:10.1016/j.cell.2014.08.009 (2014).

2 Garton, M. *et al.* A structural approach reveals how neighbouring C2H2 zinc fingers influence DNA binding specificity. *Nucleic Acids Res*, doi:10.1093/nar/gkv919 (2015).

3 Najafabadi, H. S. *et al.* C2H2 zinc finger proteins greatly expand the human regulatory lexicon. *Nat Biotechnol* **33**, 555-562, doi:10.1038/nbt.3128 (2015).

4 Smit, A., Hubley, R. & Green, P. *RepeatMasker Open-4.0*, <http://www.repeatmasker.org> (2015).

5 Hedges, S. B., Marin, J., Suleski, M., Paymer, M. & Kumar, S. Tree of life reveals clock-like speciation and diversification. *Mol Biol Evol* **32**, 835-845, doi:10.1093/molbev/msv037 (2015).

6 Gupta, A. *et al.* An improved predictive recognition model for Cys(2)-His(2) zinc finger proteins. *Nucleic Acids Res* **42**, 4800-4812, doi:10.1093/nar/gku132 (2014).
